# Multidisciplinary Management of Subclavian Artery Perforation and Complications

**DOI:** 10.7759/cureus.8009

**Published:** 2020-05-07

**Authors:** Tony Rizk, Darren Patel, Emilee Young, Vijay Ramakrishnan, Khaled Mansour

**Affiliations:** 1 Interventional Radiology, Edward Via College of Osteopathic Medicine, Blacksburg, USA; 2 Orthopedics, Edward Via College of Osteopathic Medicine, Blacksburg, USA; 3 Pediatrics, Edward Via College of Osteopathic Medicine, Blacksburg, USA; 4 Interventional Radiology, Clinch Valley Medical Center, Richlands, USA; 5 Interventional Cardiology, Clinch Valley Medical Center, Richlands, USA

**Keywords:** interventional radiology, interventional cardiology, subclavian artery perforation, distal radial artery access, percutaneous procedure, mediastinal hematoma, extrapleural hematoma, distal radial artery approach, right radial artery access, thoracic radiology

## Abstract

The radial approach to cardiac catheterization and percutaneous coronary interventions has increased in popularity due to the favorable side effect profile relative to the femoral approach. Mediastinal hematoma after radial access cardiac catheterization has scarcely been reported in the literature and, if present, the exact location of the bleed was rarely identified. In this case presentation, we describe an elective transradial coronary angiography resulting in subclavian artery perforation in close proximity to the vertebral artery, with subsequent mediastinal and cervical hematoma formation. This scenario was managed by immediate imaging of the chest after sudden deterioration raised suspicion of an adverse event during wire navigation. Formation of a mediastinal hematoma is the equivalent of retroperitoneal bleed from the femoral approach and requires rapid recognition, interdisciplinary collaboration, and endovascular management.

## Introduction

The radial approach to cardiac catheterization and percutaneous coronary intervention is favored because it is associated with less access site bleeding, definitive hemostasis, and quicker patient mobilization [1]. Disadvantages of the radial approach include radial artery injury with bleeding, pseudoaneurysm, spasm, and arteriovenous fistula formation. Rare and sometimes lethal complications of this approach include mediastinal and cervical hematomas, which occur in about 0.008% of the procedures [2]. Mediastinal hematoma after radial access cardiac catheterization has scarcely been reported in the literature, and the exact location of the bleed was rarely identified if this entity was suspected. Utilization of a straight-tipped wire when encountering vascular tortuosity should be handled with delicate care to prevent vascular dissection and perforation. These hematomas tend to occur due to the perforation of small vessels originating in close proximity to the aortic arch [2]. These proximal hematomas are particularly difficult to manage due to the inability to provide pressure hemostasis. The endovascular management of these complications using stents has demonstrated a 96.9% success rate [3]. Rapid identification and endovascular management is key to preventing patient mortality and further complications.

## Case presentation

A 75-year-old white female presented with recurrent atypical chest pain radiating to her left arm. She was on optimal medical therapy with persistent symptoms. A stress test was non-diagnostic. Therefore, she underwent a coronary angiogram using the right radial approach. Coronary angiogram revealed mid-left anterior descending artery moderate stenosis and a 3.0-millimeter drug-eluting stent was placed post-dilation with a 3.5-millimeter non-compliant balloon over a Wholey wire (Medtronic, Dublin, Ireland), which was chosen due to tortuosity encountered in the brachial artery. One brief episode of hypotension occurred and was treated with a one-time dose of intravenous phenylephrine. The patient left the cardiac catheterization lab with no immediate complications. 

**Figure 1:**
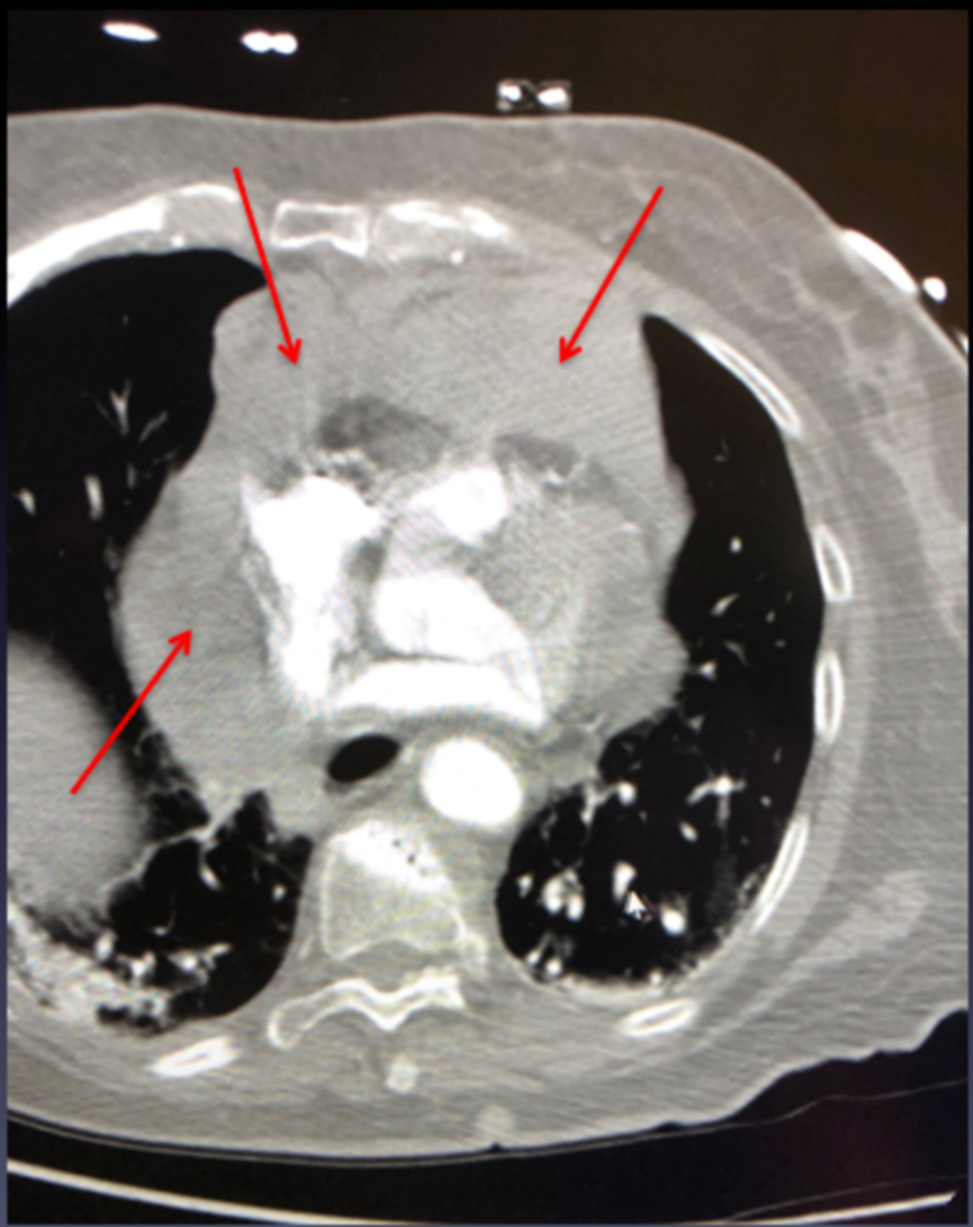
Axial contrast chest CT showing mediastinal hematomas following subclavian artery perforation.

**Figure 2:**
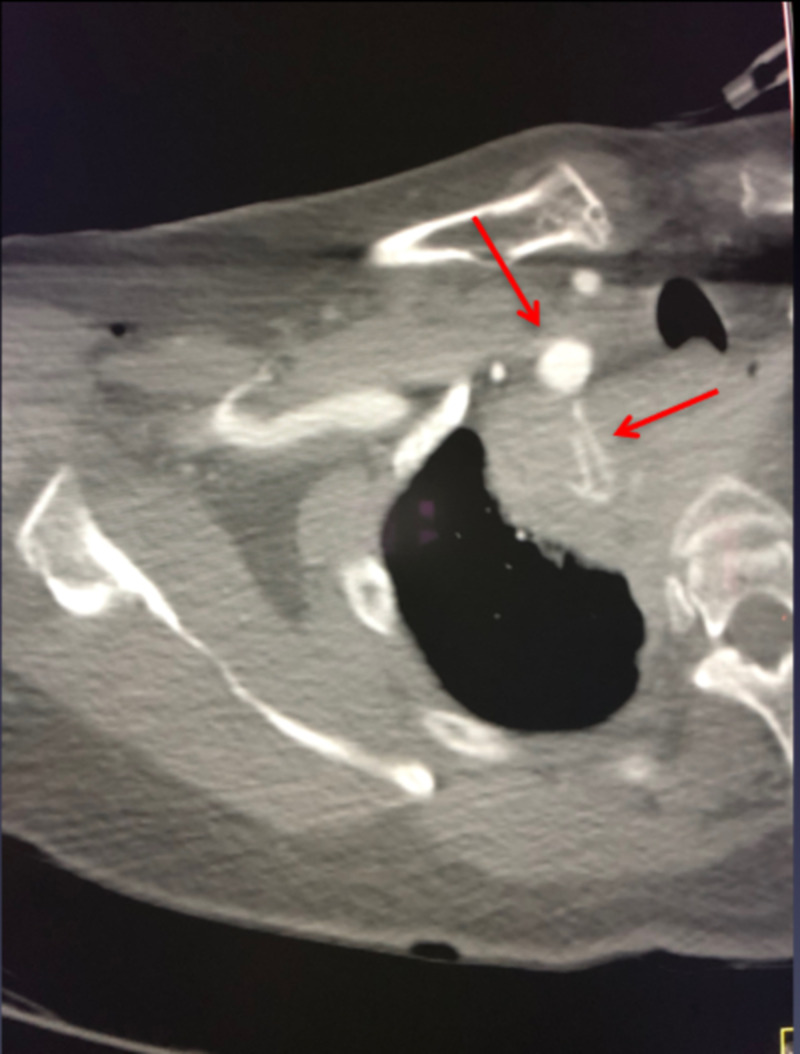
Axial CT angiogram of the chest showing active contrast extravasation from the right subclavian artery.

Thirty minutes later, the patient became hypotensive with a blood pressure (BP) of 80/60 mmHg. A fluid bolus was administered but hypotension continued to worsen, with BP dropping to 70 mmHg systolic and a heart rate of 55 beats per minute. A stat electrocardiogram showed normal sinus rhythm with a rate of approximately 55 beats per minute and low voltage as compared to baseline. Approximately 0.5 milligrams atropine and 100 micrograms of phenylephrine were administered with a brief improvement in BP. The patient then started experiencing excruciating chest pain. A bedside transthoracic echocardiography was completed with limited windows. A contrast-enhanced CT of the chest was ordered revealing a subclavian artery perforation resulting in hemomediastinum (Figures 1, 2).

**Figure 3:**
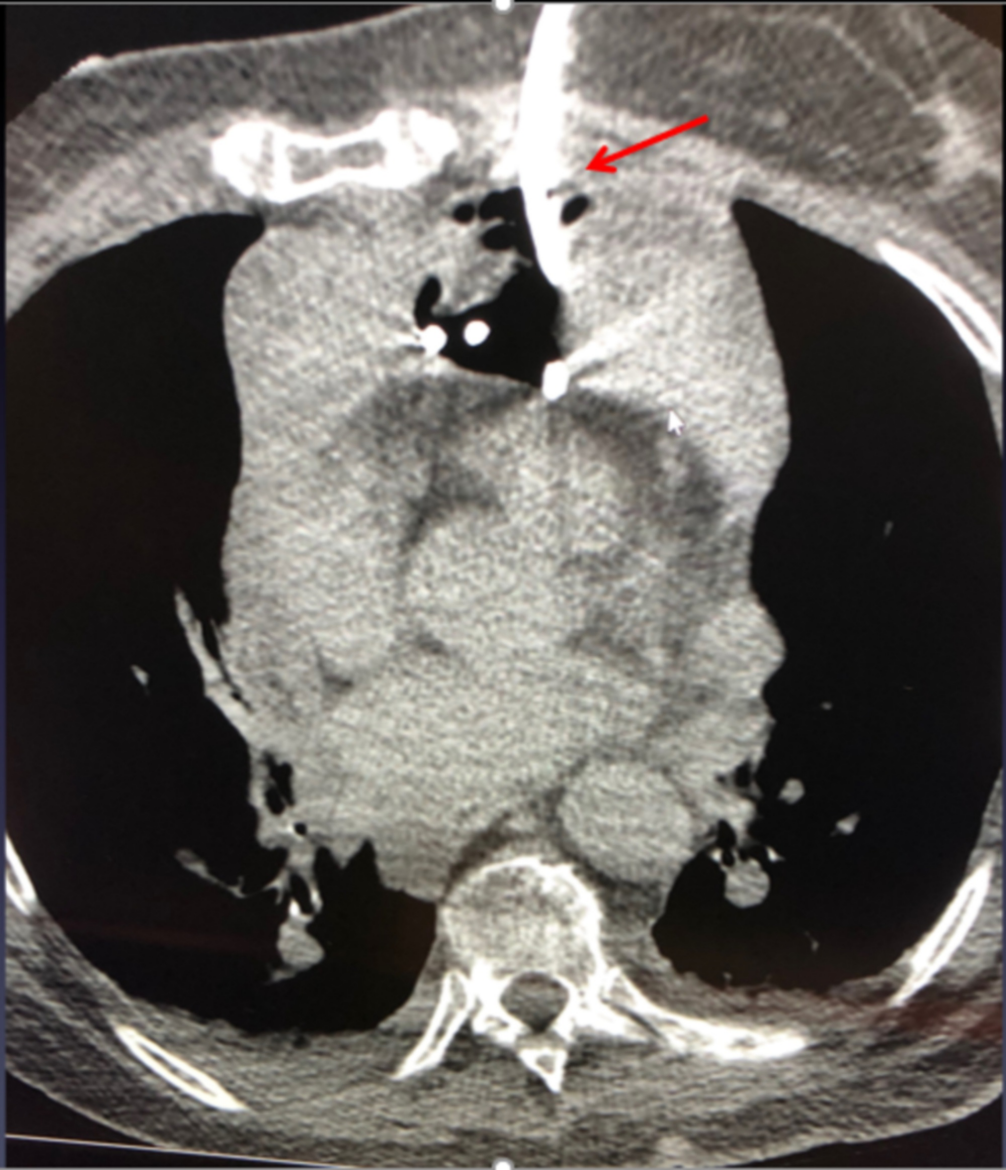
CT-guided drainage of mediastinal hematoma with an 8.5-French pigtail catheter.

Interventional radiology was consulted, and a 8.5-French pigtail drainage catheter was placed in the mediastinum using an anterior approach to prevent cardiac tamponade, taking care to avoid the internal mammary artery (Figure 3). The patient was then brought back to the catheterization lab for emergent percutaneous management. The brachial artery measured small on ultrasound; therefore, the right common femoral artery was accessed with an eight-French sheath, six-French JR4 guide. 

**Figure 4:**
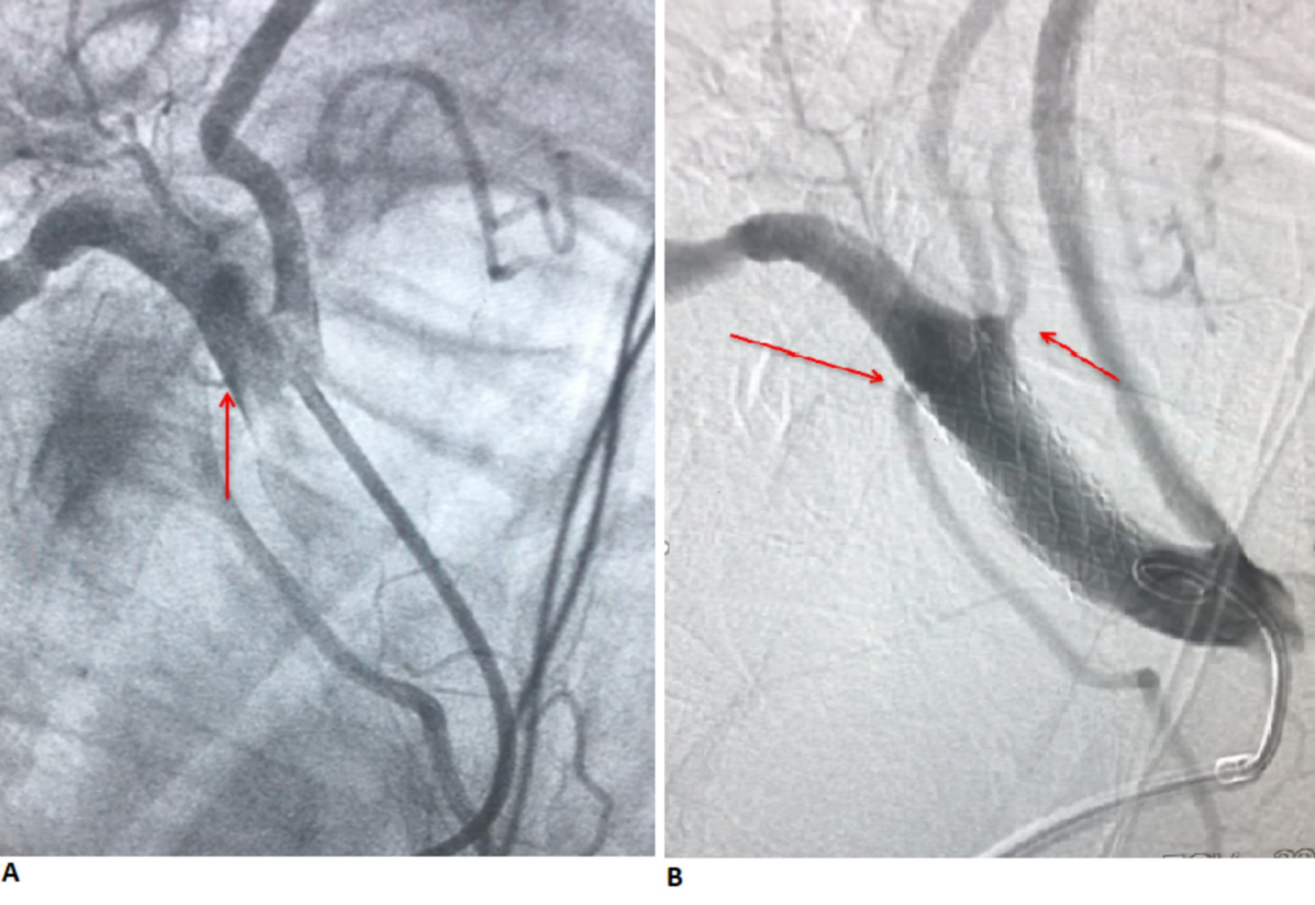
(A) Angiogram showing contrast extravasation from the right subclavian artery and the proximity of perforation to the origin of the right vertebral artery. (B) Angiogram showing stent placement with the cessation of contrast extravasation.

The right subclavian artery measured 9.0 millimeters on CT, and the site of the bleed was noted to be in close proximity to the origin of the right vertebral artery. A 10-millimeter balloon inflatable covered stent was advanced using a long sheath. The stent migrated off the balloon. Smaller balloons were used to reposition the stent, and a new 10-millimeter balloon was placed through the stent. The stent was placed with regard to the close proximity of the vertebral artery (Figure 4). The patient complained of back pain and was receiving norepinephrine 20 mcg/kg/min and dopamine 20 mcg/kg/min during the procedure. The patient received a total of four units of packed red blood cells, and the norepinephrine and dopamine infusions were stopped after six hours.

**Figure 5:**
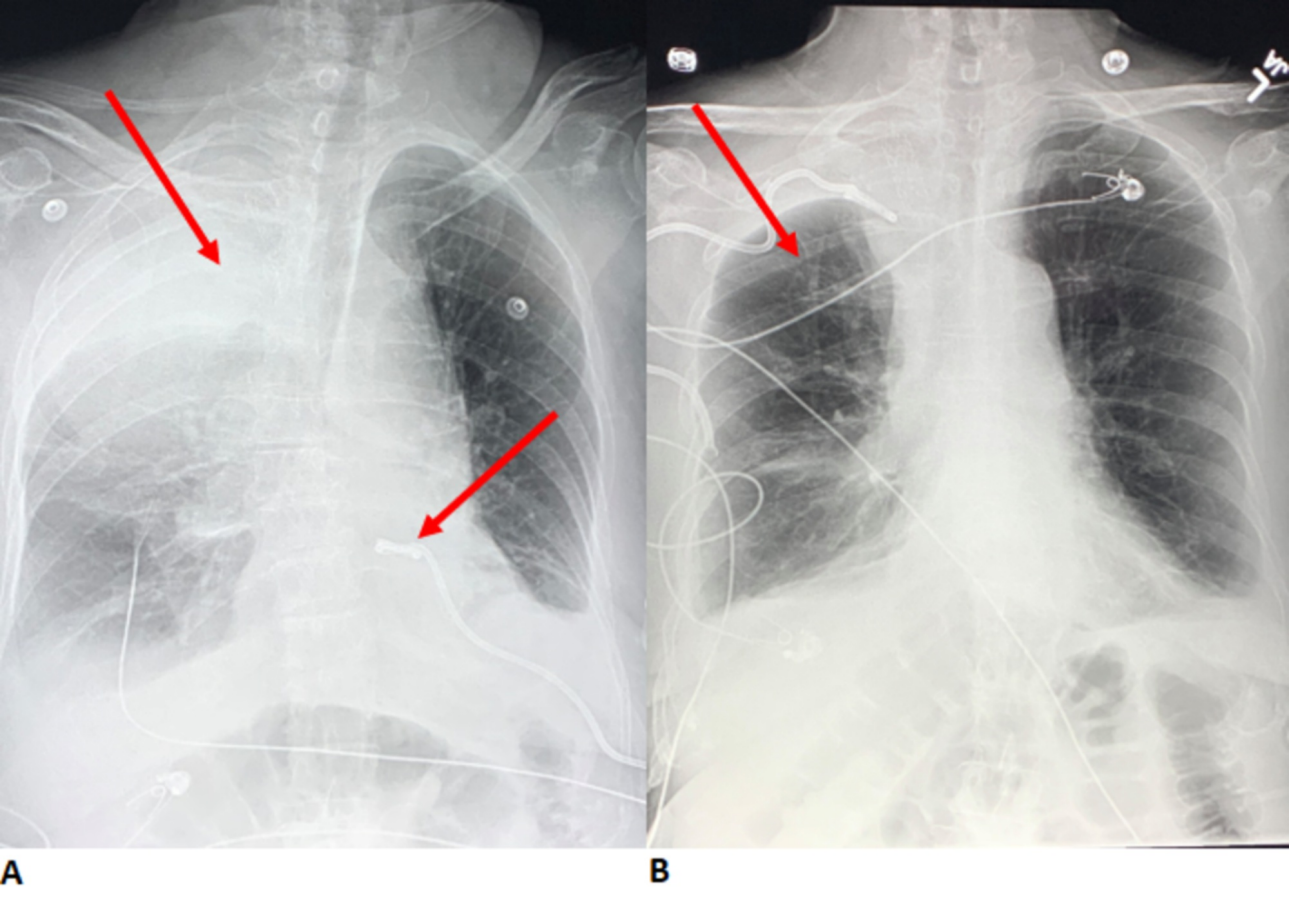
(A) AP chest radiograph with the arrow at the top pointing to a density located near the apex of the lung, consistent with a positive apical cap sign. The arrow at the bottom points to the previously placed mediastinal drain. (B) AP chest radiograph taken after CT-guided drainage of the extrapleural hematoma. AP, anteroposterior.

Ten hours after stenting, the patient started complaining of back pain and began vomiting. A chest radiograph was immediately obtained, revealing an apical cap sign with a density at the apex of the pleura (Figure 5).

**Figure 6:**
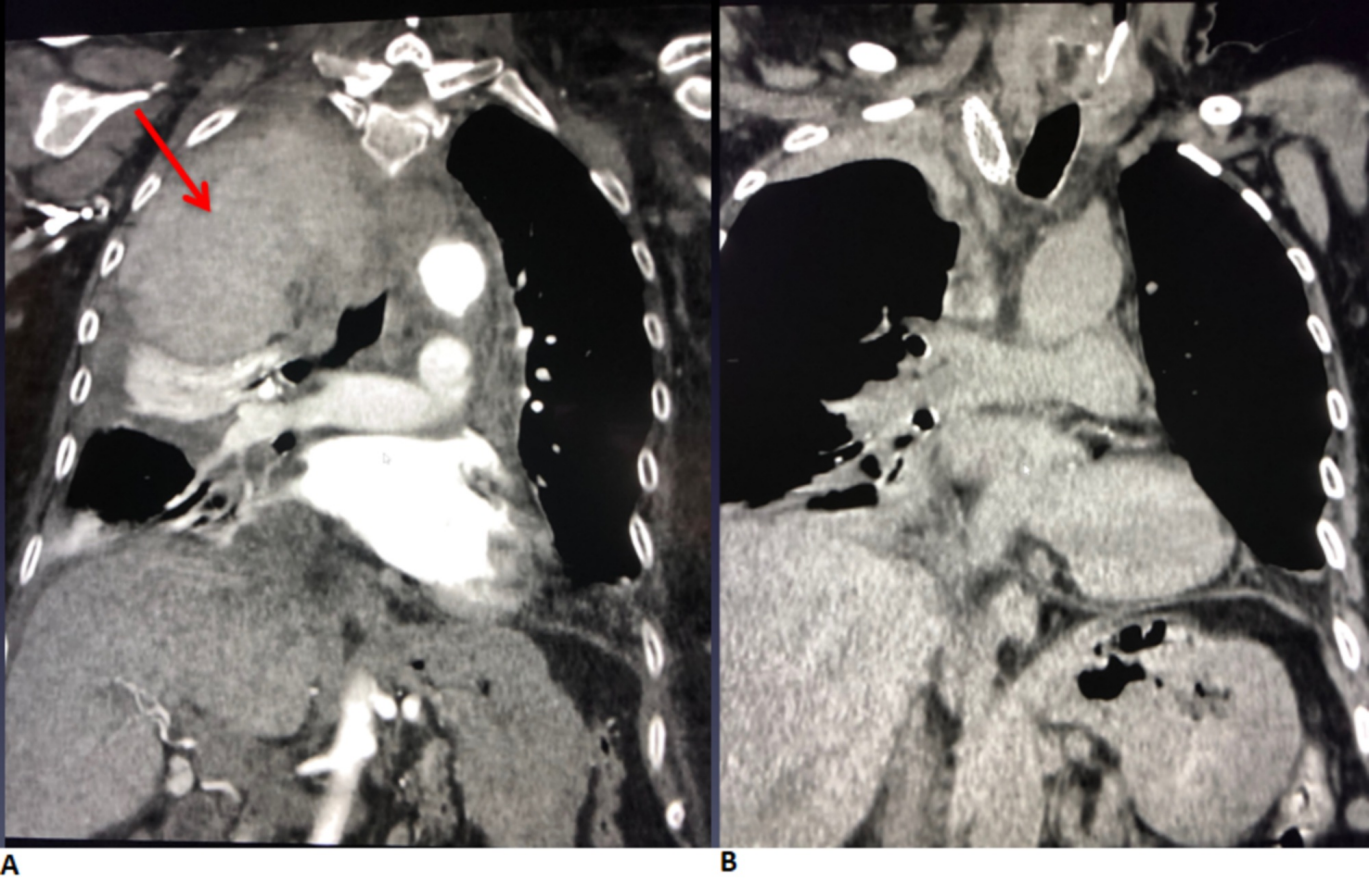
(A) Coronal CT angiogram demonstrating extrapleural hematoma compressing the right lung apex. (B) Coronal CT post CT-guided drainage of extrapleural hematoma.

On repeat CT angiography of the chest, active extravasation from the subclavian artery had ceased but an extrapleural hematoma was found. The extrapleural hematoma was drained using a pigtail drainage catheter under CT guidance (Figure 6). The patient was stable afterward and discharged home after one week. 

## Discussion

The gold standard for evaluation and management of coronary artery disease is coronary angiography. In general, the transradial approach is superior in terms of complication profile, but outcomes are more user-dependent. Proficiency in radial access requires an experienced interventionalist with technical skills typically acquired from working at a medical center with high procedural volume. The literature has shown that radial access, when compared to femoral access, is associated with a relative risk reduction in all-cause mortality, fewer major bleeding events, and major vascular complications [4]. Regardless of approach, the majority of bleeding events during percutaneous coronary intervention occurs at the site of vascular access. The superficial anatomy of the radial artery allows effective compression and rapid hemostasis [4]. The luminal caliber of the radial artery is small relative to the femoral artery, and vascular anatomic variants are more common. When these anatomic variants are encountered, such as tortuosity, utilization of a straight-tipped wire should be handled with delicate care to prevent vascular dissection and perforation. 

In rare cases, transradial access can lead to devastating central vascular perforation with hematoma formation. The first segment of the subclavian artery is anatomically defined as medial to the anterior scalene muscle, which is anterior to the cervical pleura. This anatomical relationship explains the formation of the extrapleural hematoma. The most proximal segment of the origin of the artery results in hemorrhage into the mediastinum in the event of perforation [5]. Our patient inadvertently suffered a perforation of the subclavian artery during elective coronary angiography. The patient's condition decompensated after cardiac catheterization and presented with hypovolemic shock. Subsequent immediate diagnostic imaging of the chest identified the etiology as a right subclavian artery perforation leading to mediastinal hematoma. On CT angiogram, contrast extravasation from the right subclavian artery was evident. Interventional radiology was consulted to manage the resulting hematoma by percutaneous CT-guided drainage. Angiography with iodinated contrast was then used during endovascular management to identify the leak and place an appropriately sized balloon-inflatable stent at the lesion.

We postulate that the formation of the second hematoma occurred due to the pooling of blood during the complicated stenting of the subclavian artery. The suboptimal timing of stent placement was due to stent migration in the subclavian artery. It is imperative to quickly retrieve the stent, using a snare or smaller sized balloon, in order to prevent vascular occlusion, thrombosis, and limb ischemia [6]. Balloon-inflatable stents are preferred in the management of vascular perforations, which require precise deployment [7]. Obtaining another CT scan following stent placement could have identified the extrapleural hematoma before complications clinically manifested. Endovascular treatment of iatrogenic arterial injuries by means of covered stent implants allows for rapid intervention and isolation of the lesion while maintaining adequate flow through the blood vessel, which in turn minimizes ischemic complication [8]. Endovascular management also offers a less invasive and faster alternative to median sternotomy in critically ill patients. This case effectively demonstrates the progression and management of an extremely rare and lethal complication of radial access.

## Conclusions

Transradial approach to cardiac catheterization is a commonly used procedure with a lower complication rate than the femoral approach. This case serves to outline the rapid identification and management of fatal hematomas that can complicate the transradial approach. Straight-tipped wires should be handled with caution in tortuous arteries and the subclavian artery to prevent vascular injury. In the event of perforation, rapid recognition of patient deterioration and subsequent intervention is required. The use of a balloon inflatable stent allows for rapid and precise iatrogenic arterial perforation management while avoiding the need for median sternotomy in patients that may be critically ill or hemodynamically unstable. Utilization of CT after endovascular procedures with complications may be of benefit in order to confirm the resolution of presenting lesion or identification of iatrogenic injury. Formation of mediastinal hematomas is the equivalent of a retroperitoneal bleed from the femoral approach and requires rapid recognition, interdisciplinary collaboration, and endovascular intervention.
